# Environmental Drivers and Predicted Risk of Bacillary Dysentery in Southwest China

**DOI:** 10.3390/ijerph14070782

**Published:** 2017-07-14

**Authors:** Han Zhang, Yali Si, Xiaofeng Wang, Peng Gong

**Affiliations:** 1 Ministry of Education Key Laboratory for Earth System Modeling, Department of Earth System Science, Tsinghua University, Beijing 100084, China; zhanghan11@mails.tsinghua.edu.cn (H.Z.); yalisi@mail.tsinghua.edu.cn (Y.S.); 2 Joint Center for Global Change Studies, Beijing 100875, China; 3 Center for Public Health Surveillance and Information Services, Chinese Center for Disease Control and Prevention, Beijing 102206, China; wangxf2002abc@163.com

**Keywords:** bacillary dysentery, anthropogenic environment, physical environment, logistic regression model, risk mapping, prevention and intervention

## Abstract

Bacillary dysentery has long been a considerable health problem in southwest China, however, the quantitative relationship between anthropogenic and physical environmental factors and the disease is not fully understand. It is also not clear where exactly the bacillary dysentery risk is potentially high. Based on the result of hotspot analysis, we generated training samples to build a spatial distribution model. Univariate analyses, autocorrelation and multi-collinearity examinations and stepwise selection were then applied to screen the potential causative factors. Multiple logistic regressions were finally applied to quantify the effects of key factors. A bootstrapping strategy was adopted while fitting models. The model was evaluated by area under the receiver operating characteristic curve (AUC), Kappa and independent validation samples. Hotspot counties were mainly mountainous lands in southwest China. Higher risk of bacillary dysentery was found associated with underdeveloped socio-economy, proximity to farmland or water bodies, higher environmental temperature, medium relative humidity and the distribution of the Tibeto-Burman ethnicity. A predictive risk map with high accuracy (88.19%) was generated. The high-risk areas are mainly located in the mountainous lands where the Tibeto-Burman people live, especially in the basins, river valleys or other flat places in the mountains with relatively lower elevation and a warmer climate. In the high-risk areas predicted by this study, improving the economic development, investment in health care and the construction of infrastructures for safe water supply, waste treatment and sewage disposal, and improving health related education could reduce the disease risk.

## 1. Introduction

Bacillary dysentery, a diarrhea disease caused by *Shigella*, is a considerable global health problem, especially in developing countries and underdeveloped regions with poor sanitation [1,2,3,4,5]. Facilitated by a low infectious dose [6], the disease can easily be transmitted through contaminated water, food, articles for daily use and person-to-person contact. People with poor hygienic habits are particularly vulnerable [2,7,8,9]. Socioeconomic development will affect the construction of infrastructures such as facilities for safe water supply, waste treatment and sewage disposal, which are the basic needs of life and health. However, in many underdeveloped regions the infrastructure is inadequate. People in places with a low sanitation level and lack of adequate safe water supply are especially at risk [3,7,10,11]. The seasonal variation of bacillary dysentery incidence is associated with meteorological factors [5,9,12,13,14], such as temperature, precipitation and relative humidity, which affect the growth and spread of enteric bacteria and as well as human behavior [12,13,15,16]. Many studies suggested that temperature is a positive predictive factor [5,9,13,17], while the effects of precipitation and relative humidity are inconsistent [9,10,12,14,17,18]. The divergence is probably due to the different spatial and temporal scales used in various studies. Moreover, meteorological factors may interact with social-economic environments. For instance, in poor areas that lack sanitary facilities, heavy rains can wash waste into water sources, contaminating drinking water and deteriorating environmental sanitation [10,19,20], but this kind of risk is lower in places with developed infrastructures.

In China, although the incidence of bacillary dysentery has gradually declined, a considerable health problem still exists [5,21,22]. According to the Chinese Center for Disease Control and Prevention (China CDC), there were 150,000~450,000 reported cases each year during 2005–2014, and it is one of the most commonly reported among all notifiable infectious diseases in China. The incidence of bacillary dysentery is unevenly distributed across China, with a heavy burden concentrated in the less developed western inland provinces [5,21,22].

Based on county-level data, Ma et al. [4] analyzed the spatial correlation between socio-economic factors and bacillary dysentery incidence in Sichuan Province, China. They found that gross regional product (GRP), number of beds in hospitals, medical and technical personnel were significantly negatively related to the risk of bacillary dysentery. However, they neglected physical environmental factors. Zhang et al. [5] identified some big and contiguous spatial clusters with significant high bacillary dysentery incidence in southwest China (Sichuan, Yunnan and Tibet Province) for the first time. The location and range of clusters with high incidence was almost the same over time. They conducted comparison analyses aimed at screening some potential anthropogenic and physical variables that might lead to those spatial patterns and they found that meteorological factors (temperature, precipitation and relative humidity), geographic characters (elevation, variation of elevation and slope), gross regional product and ethnic groups may have an effect. Limited by county-level health data, the disease incidence is assumed to be evenly distributed in each county in both studies. However, the effects of potential causative factors have not been quantified, and it is still not clear where exactly the bacillary dysentery risk is potentially high or low.

This study focuses in southwest China (Sichuan, Yunnan and Tibet Province) and extends the study period to 2014, aiming to understand the long-term spatial patterns of high-risk counties in this area, and to identify the risk factors that affect the spread of bacillary dysentery and quantify their associations with the disease risk, and also to predict the distribution of the high-risk regions at a finer spatial scale than the county-level health data.

## 2. Materials and Methods

### 2.1. Data Collection and Management

Data on annual bacillary dysentery incidence at district/county level in southwest China (Sichuan, Yunnan and Tibet Province) from 2005 to 2014 were obtained from the Public Health Science Data Center of China CDC. According to epidemiological features of bacillary dysentery and published research findings [4,5], we collected 12 kinds of anthropogenic and physical environmental data that were potentially associated with bacillary dysentery. 

Multiyear monthly air temperature data and GRP data were collected from the Global Change Research Data Publishing & Repository [23]. Monthly precipitation and relative humidity were obtained from the China Meteorological Data Sharing Service System [24] and were interpolated into raster surfaces using an inverse distance weighting (IDW) technique. Data on ethnic group distribution were obtained from the *Communist China Map Folio* [25]. SRTM (Shuttle Radar Topography Mission) 90 m digital elevation data were collected from the Consultative Group for International Agricultural Research Consortium for Spatial Information [26]. River and lake data were acquired from the National Geometrics Center of China [27]. Terrain data were obtained from the *Geographic Atlas of China* [28]. Distributions of forest and farmland were extracted from FROM-GLC-agg, an improved 30 m global land cover dataset [29,30,31,32,33,34,35]. The land cover map was based on Landsat TM/ETM+ (Thematic Mapper/Enhanced Thematic Mapper) images and the MODIS (Moderate Resolution Imaging Spectroradiometer) time series images around 2010. Data on the numbers of hospital beds in each district/county in 2010 were extracted from *China County Statistical Yearbook* [36]. The environmental data used for statistical analyses in this study are summarized in Table 1.

With the obtained data, we derived 20 variables for subsequent analyses which are summarized in Table 2. Ethnic group, number of hospital beds, forest coverage and terrain types were categorical variables, Ethnic-2 represented the Tibeto-Burman group while Ethnic-1 represents other groups. An ethnic group is included because the high-risk counties are dominated by the Tibeto-Burman people [5] and these people have been shown to be more susceptible to bacillary dysentery than others due to unsanitary living conditions and unhygienic living habits [8,37,38]. Terrain-2 represents mountain land while Terrain-1 represents other types of terrain (mainly basin, hill and plateau). Terrain type is included because the high-risk counties are located in mountainous areas [5]. According to the results of Zhang et al. [5], the Tibeto-Burman people in mountainous areas are at high risk. Therefore, a composite variable EthnicTerrain was created to investigate potential linkages between ethnicity and terrain types. EthnicTerrain-2 represents Tibeto-Burman people in Mountain areas while EthnicTerrain-1 represents other situations. The number of hospital beds represents the level of government investment in local medical and public health, and is negatively associated with the incidence of bacillary dysentery [3,4]. Hospital bed data are county-level statistics, but to use them directly as a continuous variable would cause autocorrelation. In order to facilitate the analyses, data on county-level hospital beds were discretized into four classes using the quantile method. Detailed information on discretized hospital beds is shown in Table 2.

Elevation, its variation and slope were derived from DEM (Digital Elevation Model). Elevation variation was utilized as a description of macroscopic relief amplitude, which was calculated as the standard deviation of elevation in a 10 km × 10 km basic unit in this study. Elevation variation was introduced because the high-risk counties had a significantly greater variation of elevation compared with other counties [5], and places with great relief amplitude are usually mountain regions where the population is extremely poor [39]. Slope represents the degree of steepness, which are microscopic characters of topography. People tend to reside in relatively flat places (such as basin, river valley etc.) rather than steep hillside. Annual average, summer and winter temperature/precipitation/ relative humidity was aggregated from the meteorological data we obtained.

In southwest China, the population is sparsely distributed. Obviously, uninhabited places should not be recognized as high risk areas, even though such places may also provide appropriate environmental conditions for the spread of bacillary dysentery. Farmland is a direct mark of human presence on Earth. Places with farmland are typical rural areas, where the water supply and sanitation infrastructure is poorer compared with urban areas. Moreover, farmers were found to be more prone to bacillary dysentery due to the low-level sanitary conditions of their living and working environment and lack of health-related knowledge [3,4,8,40]. Thus, distance to farmland was included because the places where rural people live and work are typically not far from farmland. Surface water such as rivers and lakes are vital for human’s living and working. People in mountainous areas usually reside near water bodies. In addition, water is an important medium of bacillary dysentery transmission. Therefore, distance to water bodies was introduced in the model. The distance was calculated using Cost Distance tool in ArcGIS (Version 10.2, ESRI Inc., Redlands, CA, USA), which can compute the least accumulative cost distance for each point (pixel) to the nearest objects over a cost surface. The inverse of cosine slope was set as cost surface to calculate the approximate surface distance. Forest was used as an exclusion mark of human residential areas in the model, as forested areas are unlikely to contain a densely populated area.

Before analyses, all the environmental data was transformed to raster file with 3 arc second (approx. 90 m) spatial resolution under WGS-84 geographic coordinate system. We adopted Albers projection (central meridian: 95°E; standard parallels: 25°N, 35°N), a double standard-parallels, equal-area, conic projection to reduce the distortion in our study area.

### 2.2. Ethical Statement

Data on bacillary dysentery used in this study were anonymous and de-identified and were aggregated by administrative regions. No informed consent was required as no individual-level analysis was performed in this study. The data were stored in a password-encrypted file in a single personal computer. Authors are not authorized by the data providers to disseminate the data nor to generate copies. Thus, no approval from Institutional Review Board or equivalent ethics committee was needed.

### 2.3. Statistical Analysis

According to the first law of geography “everything is related to everything else, but near things are more related than distant things” [41]. However, the incidence of bacillary dysentery in southwest China might fluctuate spatially due to the small population base, and this indicated the presence of biases [42]. To reduce the fluctuation, a Spatial Empirical Bayes Smoothing [43] in GeoDa software (Version 1.8, The University of Chicago, Chicago, IL, USA) was first applied to smooth the origin incidence data. Based on the smoothed county-level incidence, hot spot analysis, (which calculated the Getis-Ord Gi* statistic [44]), was applied to identify the high-risk districts/counties using ArcGIS software. The conceptualization of spatial relationships adopted the default fixed distance band method. To be a statistically significant hot spot, a county should not only have a high bacillary dysentery incidence but also be surrounded by other counties with a high incidence [44].

**Figure 1:**
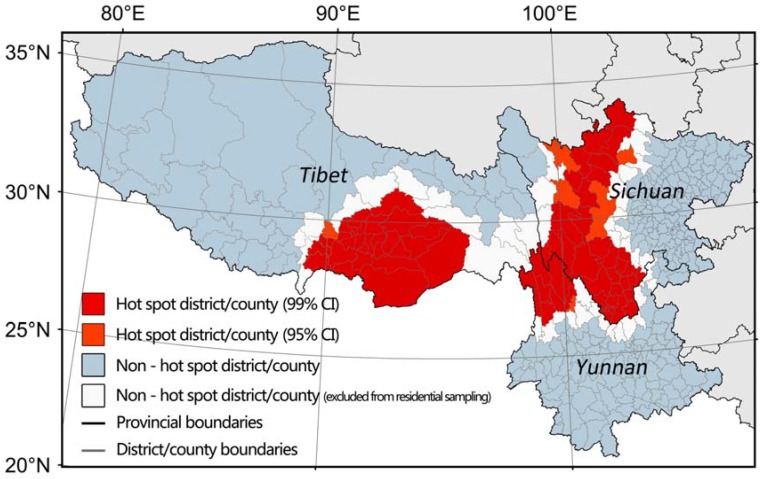
Distribution of hotspot districts/counties in southwest China from 2005 to 2014. (The white counties are indicated because they were excluded from residential sampling due to their uncertain status according to the hot spot analysis algorithm).

To build a spatial distribution model, presence and absence samples were needed for model training. Individual records of bacillary dysentery cases with detailed locations were not available for privacy reasons, thus presence samples were generated from residential sites at village level. We assumed that residential sites in the hotspot counties were at high-risk and therefore they could be selected as presence samples. Absence samples were consisted of two parts. One part was randomly generated from non-residential areas throughout southwest China (i.e., glacier, swamp, forest, steep hillside, barren land and others) and the other was obtained from residential areas in non-hotspot counties. Each part accounted for about 50% of the absence samples. Non-residential samples were then checked one by one on Google Earth to make sure they did not fall into residential areas in hotspot regions or surrounding counties (white in Figure 1). The white counties were excluded for residential sampling, because the status of these counties was uncertain according to the hot spot analysis algorithm [44].

We randomly selected 1270 presence samples, 10% (127 samples) of which were randomly extracted for independent validation, and the rest 1143 samples were used for model training. A total of 5127 absence samples were selected, among which 127 samples were randomly extracted for independent validation, and the remaining 5000 samples were used for model training. The Geospatial Modeling Environment (GME) tool embedded in ArcGIS was utilized to generate the random samples. In order to maintain the effectiveness and heterogeneity of the samples, the distance of neighboring samples was at least 1 km.

Logistic regression analyses were then applied to examine the relationship between the environmental variables and the bacillary dysentery risk. Mcpherson et al. [45] demonstrated that optimal models developed from logistic regression had intermediate prevalence and large sample sizes. We therefore utilized a bootstrapping procedure with all the 1143 presence locations and 1143 absence locations randomly selected from the 5000 absence locations. The procedure was repeated 1000 times, creating 1000 training subsets for model training (Figure S1 shows an example of a training subset).

Univariate logistic regression analyses were carried out to examine the linear and quadratic effects of each variable. The effect of each variable was evaluated by mean values of odds ratios (OR), *p*-value, pseudo *R*^2^, Akaike’s information criterion (AIC), AUC and Kappa, which were calculated using 1000 different training subsets. If OR is greater than 1, then an increasing value of the variable is a risk factor, otherwise it is a protective factor. If OR approximates to 1, the effect of the variable is limited. Pseudo *R*^2^ and AIC are measures of the relative quality of statistical models for a given set of data. The higher *R*^2^ or lower AIC, the better the model. The Receiver Operating Characteristic (ROC) curve is a graphical plot that illustrates the performance of a binary classifier system as its discrimination threshold varies. AUC and Kappa are quantitative indicators of the model performance. In this stage, variables with *p*-value ≥ 0.1 were removed for further analyses [46,47]. Autocorrelation and multi-collinearity were assessed by examining Moran’s I [48] (Moran, 1950) and the variance inflation factor (VIF) [49], respectively. Variables with relatively high spatial autocorrelation (absolute value of Moran’s I ≥ 0.5) were dropped [46,47]. Variables with relatively high collinearity (VIF > 10) were removed [50].

Besides AIC, the necessity for a quadratic form of a variable could also be tested by using the ANOVA likehood ratio test method, which determines the difference between the model with and without quadratic terms. *p*-value < 0.05 indicates there is a significant difference between the two models. Whether or not to include a quadratic form of a variable was also determined by the prior knowledge on bacillary dysentery. Many studies have observed an almost linear positive relationship between bacillary dysentery and temperature [9,13,14,17]. Moreover, microbiological studies have indicated that the optimal temperature for *Shigella* is 37 °C [51], which is higher than the highest monthly average maximum temperature observed in Sichuan (33.4 °C), Yunnan (35.6 °C) and Tibet (26.1 °C) during the study period. Therefore, we excluded the quadratic term of temperature in our models. However, previous reports about the effects of precipitation and relative humidity are inconsistent [9,10,12,14,17,18]. Thus, we included quadratic term of precipitation and relative humidity to test if there is non-linear relationship between them and bacillary dysentery in our study area.

Multiple backward stepwise logistic regressions were executed using the variables selected by univariate analyses. This backward stepwise process was repeated 1000 times utilizing different training subsets. The frequency of each variable being selected and the mean *p*-value of each variable were calculated. Variables yielding non-significant effects (Mean *p*-value ≥ 0.05) were not considered for selection.

Multiple logistic regressions were finally carried out using the remaining significant variables. The process was also repeated 1000 times using different training subsets. The mean values of coefficients, OR, *p*-value, AIC, AUC, optimal threshold of ROC curve and kappa were calculated and utilized as indicators of model performance. The risk map was then predicted based on the model and the probability was calculated as:

$$P=\frac{\exp\left(\beta_{0}+\beta_{1}x_{1}+\beta_{2}x_{2}+\ldots +\beta_{n}x_{n}\right)}{1+\exp\left(\beta_{0}+\beta_{1}x_{1}+\beta_{2}x_{2}+\ldots +\beta_{n}x_{n}\right)}$$

where, $x_{n}$ are the key environmental factors, and $\beta_{n}$ are their coefficients in the model. *P* is the probability of disease risks, a continuous number from 0 to 1. Places with *P* higher than the optimal threshold are high-risk, while places with *P* lower than the threshold are low-risk. Logistic regression analyses were carried out using R software (Version 3.2.2, R Core Team, Vienna, Austria) [52].

## 3. Results

The distribution of multiyear hotspot districts/counties is shown in Figure 1. In Figure 1, the red and orange districts/counties are hot spots with confidence interval above 95%, while the white and blue ones are not. In Sichuan and Yunnan, high-risk counties are distributed along the terrace from the eastern lowland to the western Tibetan Plateau. In Tibet, high-risk counties are located in the southeast corner of Tibet, which is the only entrance from the plains of India to the Tibetan Plateau at ground level. The hotspot regions are typical mountain lands and the location and range of hotspot regions are almost the same during the study period (Figure S2). The results of the hot spot analyses guided sampling work and following analyses.

In univariate logistic regression analyses, significant positive associations (*p* < 0.05) were found between dysentery risks and T_Ann/Sum/Win, Elevation, ElevationSD, RH_Sum. Significant negative associations (*p* < 0.05) were found between dysentery risks and DisFarm, DisWater, GRP, Slope, PR_Win, RH_Win. PR_Ann/PR_Sum and RH_Ann had no significant linear association with dysentery risks. Including the quadratic form of ElevationSD, DisFarm, DisWater, GRP, PR_Ann/Sum/Win, RH_Sum and Slope in the regression model would not greatly improve its performance.

In southwest China, temperature varies vertically mainly due to terrain. Air temperature is negatively associated with elevation. Elevation was excluded as a confounding variable for further analyses, because temperature was the key factor that affected the growth and spread of enteric bacteria [5,9,13,17]. Empirically, most infection of bacillary dysentery occurs in summer, thus winter meteorological variables were not included. There was an obvious linear correlation between T_Ann and T_Sum, and we selected T_Ann due to the lower AIC value. Categorical variables were all significantly correlated to the outcome that we were interested in. Ethnic and Terrain were excluded because this information was contained in variable EthnicTerrain.

After univariate analyses, diagnostics of autocorrelation and multi-collinearity and the stepwise selection, ten variables (i.e., DisFarm, DisWater, GRP, EthnicTerrain, Forest, Slope, ElevationSD, RH_Ann, AT_Ann and BedNum) were finally selected as inputs for the final multiple logistic regressions. Using the bootstrapping strategy, 1000 models were fitted and the mean value of each output parameter was calculated. Table 3 demonstrates the result of the multiple logistic regressions. The first column lists the variables included in the model. The odd ratio is the natural exponential of coefficients.

Proximity to farmland or water bodies, lower GRP, higher environmental temperature, medium relative humidity, smaller slope, greater elevation variation, the Tibeto-Burman people living in mountainous areas, non-forest land and fewer hospital beds were identified as the risk factors, which would increase the risks of bacillary dysentery infection.

**Figure 2:**
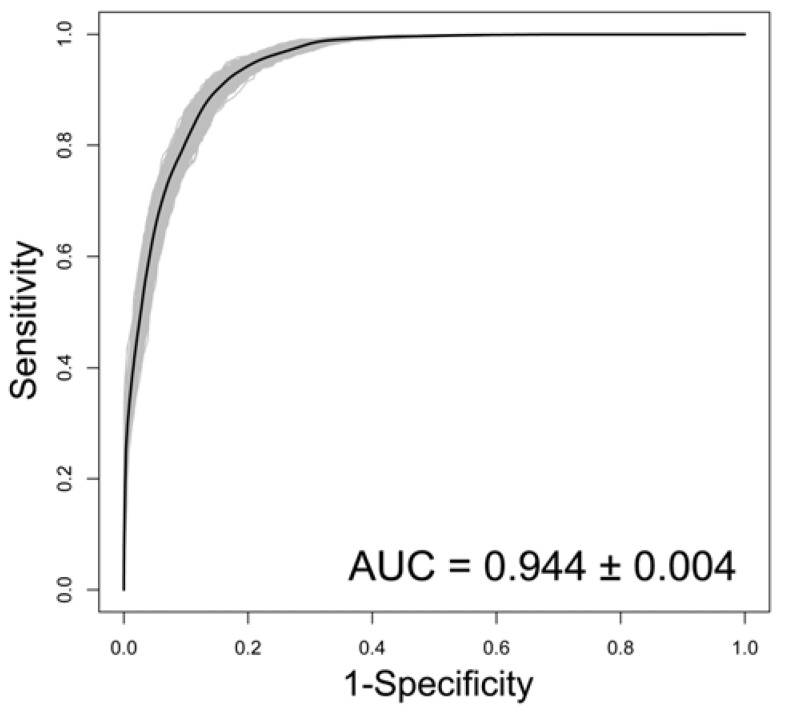
ROC curves of the predictive power of the multiple logistic regression models on the risks of bacillary dysentery in southwest China. (Gray lines: ROC curves of the 1000 fitted models; Black line: mean ROC curve of the 1000 fitted models).

**Figure 3:**
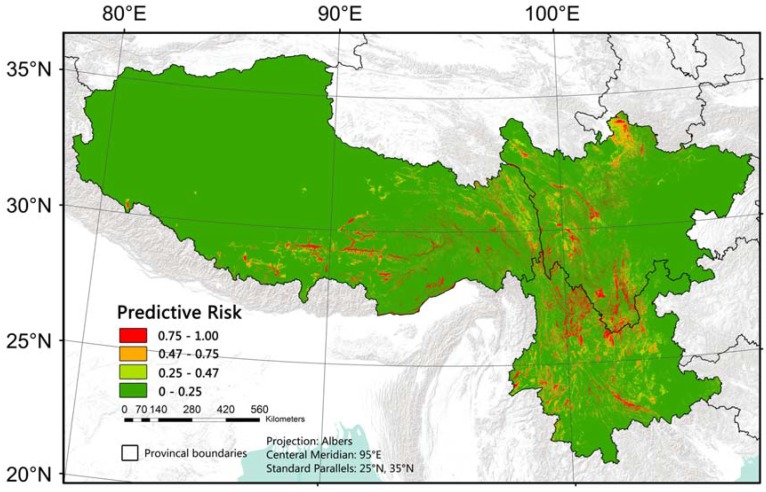
Predictive risk map of bacillary dysentery in southwest China.

The predictive ability of the model was good. The AUC was 0.944 and kappa was 0.75 (Figure 2) and the independent validation indicated that overall accuracy was 88.19% (Table 4). The optimal threshold of the model was 0.47. Based on the established model and variables selected, we generated a predictive risk map of bacillary dysentery in southwest China (Figure 3). This map demonstrates the probability that a place is at high risk of bacillary dysentery. The possibility of the red regions possessing a long-term high incidence rate of bacillary dysentery is over 75%.

## 4. Discussion

Hotspot counties were mainly located in the south-north oriented mountains in the mid-west of Sichuan Province, northwest corner of Yunnan Province and southeast part of Tibet, all of which are mountainous lands. The distribution of hotspot counties identified in this study was consistent with the spatio-temporal clusters recognized in previous studies [4,5]. The long-term spatial pattern hinted at the existence of risk factors. For the first time, this study has conducted comprehensive analyses and identified ten environmental factors (i.e., distance to farmlands, distance to water bodies, GRP, Tibeto-Burman people living in mountain areas, forest, slope, elevation variation, annual relative humidity, annual air temperature and number of hospital beds) that were associated with bacillary dysentery in southwest China, and predicted the bacillary dysentery risk based on these factors.

The incidence of bacillary dysentery is negatively associated with socio-economic status worldwide [1,4,5,9,11]. Socio-economic status includes many dimensions, such as economy, education, health care, infrastructure and even ecological factors like natural resources, disaster and topographic conditions [39]. GRP is a direct indicator of economy. In this study, a higher GRP was identified as a protective factor while a lower GRP was a risk factor, which agrees with previous studies [1,4,5,9,11]. Better economic status is usually associated with better living conditions, environmental sanitation, food and water supply and education, which can help reduce the spread of bacillary dysentery.

This study included elevation variation as an indicator of relief amplitude and found that elevation variation was an effective supplement of economy data, and that the greater the variation of elevation the greater risk factor is. Terrain restricts the distribution of resource and energy, and regions with large relief amplitude (i.e., large mountainous areas) are usually associated with areas of extreme poverty, poor education and inadequate infrastructure construction (i.e., safe water supply, waste treatment and sewage disposal), and are also prone to geographic disasters [39], which facilitate the spread of bacillary dysentery. Moreover, five of the 14 national contiguous extreme poverty regions were located in our study area [53], all of which were mountainous lands with wide variations of elevation.

Our model indicated that the number of hospital beds was negatively associated with dysentery risk, which corresponded with Ma et al. [3,4]. In this study, we found that the risk for BedNum-4, BedNum-3 BedNum-2 counties was more than 20, 10 and 2 times respectively of BedNum-1 counties (Table 3). The number of hospital beds is an important indicator of local medical conditions. Better medical conditions might helpful for the reducing of disease transmission [3,4].

Hotspot counties identified in this study were found to be mostly inhabited by Tibeto-Burman people. Published studies found the Tibeto-Burman people were more susceptible to bacillary dysentery than other ethnicities. The situation seems even worse if Tibeto-Burman people reside in underdeveloped mountainous areas [5]. Our model indicated that the risk of Tibeto-Burman people who reside in mountainous areas to be 3.06 times than that of other ethnic groups or Tibeto-Burman people elsewhere (Table 3). This may not only associated with their unsanitary living conditions, but also to their cultural and living habits, such as the intake of raw food or water, eating with their hands instead of using tableware and some particular food production methods [8,37,38].

As the human is generally susceptible to bacillary dysentery, we also concentrated on the environments where people live and work. In this study, distance to farmland, distance to water bodies and slope were found to be negatively associated with the risk of bacillary dysentery. Farmland is a mark of human existence and also an indication of a rural area. Farmers were found to be more susceptible to bacillary dysentery than people engaged in other occupations, which was due not only to the low-level sanitary conditions of their living and working environment, but also to a lack of health related knowledge [3,4,8,40]. Generally, farm houses are adjacent to, or even part of the related farmland, while rural people who engage in other occupations live further away from farmland, and urban people live furthest from farmland. Because surface water such as rivers and lakes are vital for living and working, people in mountainous areas usually reside near to water bodies. People living near to rivers are more likely to use surface water, which might expose them to more sewage compared to people who live far away from rivers [54], as water is a convenient medium for *Shigella* transmission. Slope is a microscopic character of terrain that determines distribution of population, as people tend to reside in relatively flat places (such as basin, river valley etc.) rather than steep hillside.

Higher annual temperature was identified as a risk factor for higher bacillary dysentery incidence. Within the temperature range suitable for human survival, higher temperature will facilitate the growth and spread of *Shigella* [51]. Temperature not only affects the activity of pathogens but also the distribution of human beings. In southwest China, temperature varies vertically mainly due to terrain. People mainly concentrate in basins or river valleys where elevation is relatively low and the climate is milder instead of high frigid places [55]. The results suggest that relative humidity may also affect the spread of bacillary dysentery in southwest China, and areas with medium annual relative humidity (50%) are at the highest risk. The underlying reason might be that low relative humidity does not facilitate the survival and spread of *Shigella* [56], while high relative humidity (>50%) normally comes with more precipitation which can purify the environment [14]. Few studies have examined the relationship between humidity and rainfall and reported inconsistent findings. One study in Sub-Saharan Africa found that shortage of rainfall increased the prevalence of diarrheal diseases [57]. One study observed a positive association for humidity and precipitation with bacillary dysentery in Shenyang city, China [13], whereas no significant effect of relative humidity and rainfall was detected in Jinan city and Shenzhen city, China [58]. The discrepancy might be due to different socioeconomic development, human habits and population characteristics in various regions.

**Figure 4:**
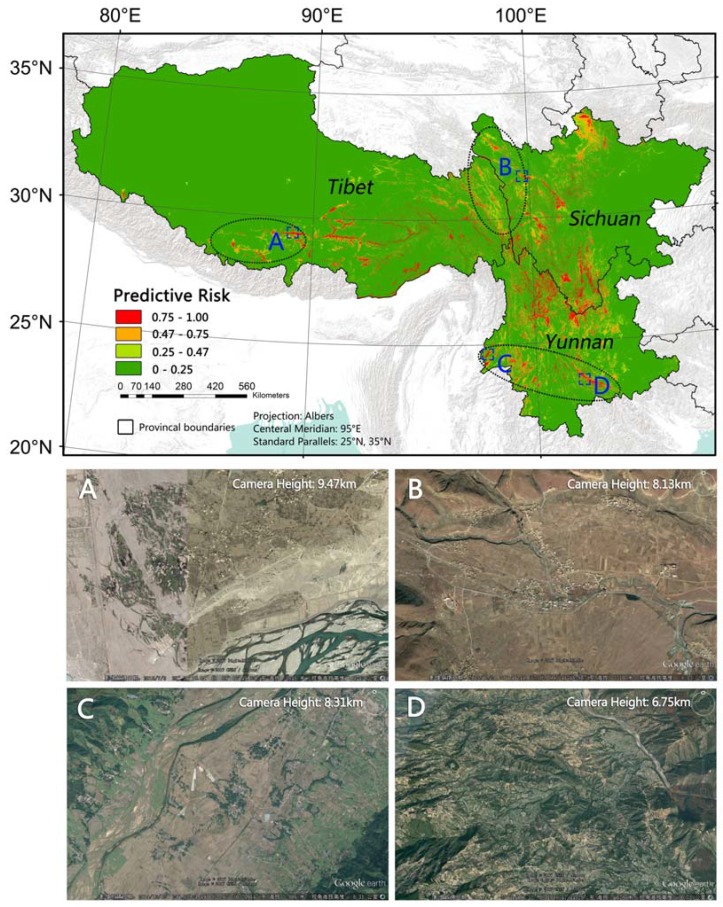
Predicted high-risk patches in non-hotspot regions. (**A**–**D** are four examples of predicted high-risk patches in non-hotspot region. Residents in site **A**, **B** and **C** live in river valleys, residents in site **D** live on rugged lands. The four images are Google Earth images in corresponding sites showing the poor living conditions).

The predictive risk map demonstrates the probability of an area being at a high risk of bacillary dysentery. Different from traditional disease mapping that is based on polygonal administrative units, this map provides a detailed downscaling view of its spatial pattern, which is more realistic than the evenly distributed risk in a county. AUC, Kappa and the independent validation indicated that the model performed well. We overlaid the hotspot map on the predicted risk map and found that the distribution of high-risk areas in predicted map are generally within the range of hotspot regions. We found there are a few big and obvious red patches (predicted to be of high risk) in non-hotspot region between the Sichuan hotspot cluster and the Tibet cluster, in non-hotspot on the west of the Tibet hotspot cluster, and in the mid-south of Yunnan (See black circles in Figure 4). These regions are remote rural areas. Residents there live around river valleys or even rugged lands. Living conditions there are very poor, which can be seen from Google Earth (Figure 4). The high-risk patches outside the hotspot regions might be omitted by passive disease surveillance system [59], or the detailed patterns were hidden when aggregated to county-level administrative regions. Therefore, comparing to county-level mapping, the predicted risk map may provide higher quality not only in fineness, but also in accuracy.

**Figure 5:**
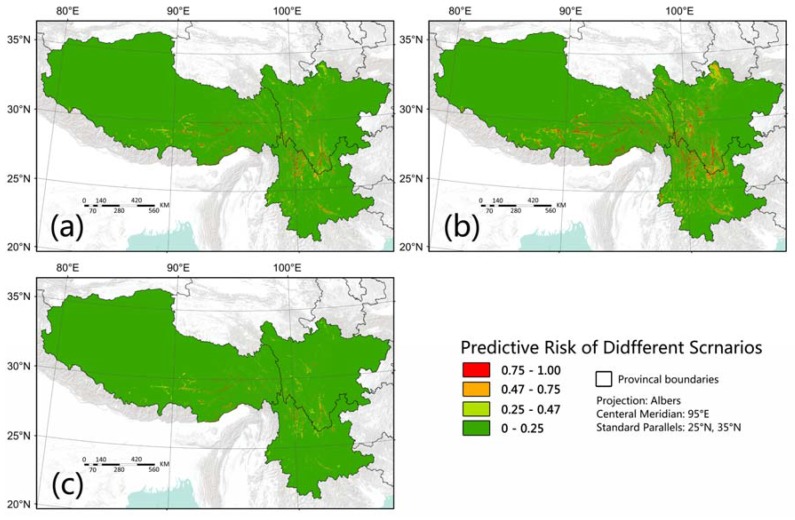
Predicted risk of bacillary dysentery in different hypothetical scenarios. (Scenarios (**a**): GRP per square kilometer of the under developed places are improved to the contemporaneous level of that in the urban area of Lhasa. Scenarios (**b**): the number of hospital beds rise by one level. Scenarios (**c**): both (**a**) and (**b**)).

To reduce the risks of bacillary dysentery in southwest China, we suggest improving the level of economic development and increasing investment in health care. Figure 5 shows some hypothetical scenarios. Figure 5a shows a scenario that GRP per square kilometer in the under developed places were improved to the level of that of the urban area of Lhasa (the capital city of Tibet). Figure 5b shows a scenario that the number of hospital beds was raised by one level. Figure 5c shows a scenario that both GRP and the number of hospital beds were improved. A significant reduction of bacillary dysentery risk in southwest China was found in all hypothetical scenarios (Figure 5). Unlike GRP and BedNum, which can be easily supposed and controlled in the model, variables such as DisFarm, EthnicTerrain and ElevationSD are superficially invariant. But their meanings, which have been discussed in previous paragraphs, suggest some additional ways to improve public health status in these regions. Investment on construction of infrastructures for safe water supply, waste treatment and sewage disposal should be increased in poor areas. Health related education should be strengthened among farmers and the Tibeto-Burman people with respect to their traditional cultures.

The design and findings of this study are reasonable, but some limitations should be noted. Due to lack of more detailed health data, the sampling work in this study was based on previous statistical analyses and reasonable assumptions. Were more detailed health data available, the model and prediction could be improved. We could not know whether the overestimation in some places in this study was due to omission in official data or defects in environmental data or model building. A more targeted effort is needed in future study. Categorization of hospital bed data would exaggerate the difference of values near the thresholds. Therefore, a better form of variable transformation or alternative variable is needed.

## 5. Conclusions

Hotspot districts/counties were mainly located in the south-north oriented mountains in the mid-west of Sichuan, the northwest corner of Yunnan and the southeast of Tibet, all of which are mountainous areas with great relief amplitude. Higher risks of bacillary dysentery are associated with under developed social economic conditions, close to farmlands or water bodies, higher environmental temperature, medium relative humidity and distribution of the Tibeto-Burman people, which may be related to their living habits. High-risk places are mainly located in mountainous areas where the Tibeto-Burman live, especially in basins, river valleys or other flat places with relatively lower elevation and milder climate. Improving economic level and investment in health care and construction of infrastructures for safe water supply, waste treatment and sewage disposal, strengthening health related education in the high-risk places, as shown in our modeling, could reduce the disease risk. Findings from this study could provide useful information for better interventions and public health planning in southwest China.

## Figures and Tables

**Table 1:** Summary of environmental datasets used in this study.

Category	Description of Datasets	Format	Resolution
Anthropogenic environmental data	GRP (gross regional product)	Raster	1 km
	Ethnic group	Polygon	
	Number of hospital beds	Polygon	
Physical environmental data	DEM (digital elevation model)	Raster	90 m
	Temperature	Raster	1 km
	Precipitation	Point	
	Relative humidity	Point	
	Rivers	Polyline	
	Lakes	Polygon	
	Forest	Raster	30 m
	Farmland	Raster	30 m
	Terrain type	Polygon	

**Table 2:** Summary of environmental variables used for analysis in this study.

Category	Description of Variables	Type	Abbreviation	Unit
Anthropogenic environmental variables	GRP per 1 km^2^	Continuous	GRP	10 million yuan/km^2^
	Ethnic group (2: Tibeto-Burman; 1: Others)	Categorical	Ethnic	No unit
	Number of beds in hospitals (4: fewer than 204 beds; 3: 204–668 beds; 2: 669–1678 beds; 1: more than 1679 beds)	Categorical	BedNum	No unit
Physical environmental variables	Elevation	Continuous	Elevation	100 m
	Elevation variation	Continuous	ElevationSD	100 m
	Slope	Continuous	Slope	°
	Temperature (Annual/Summer/Winter)	Continuous	T Ann/Sum/Win	°C
	Precipitation (Annual/Summer/Winter)	Continuous	PR Ann/Sum/Win	mm
	Relative humidity (Annual/Summer/Winter)	Continuous	RH Ann/Sum/Win	%
	Distance to water bodies	Continuous	DisWater	km
	Distance to farmlands	Continuous	DisFarm	km
	Forest coverage (2: Forest; 1: Non-forest)	Categorical	Forest	No unit
	Terrain type (2: Mountain land; 1: Others)	Categorical	Terrain	No unit
Interaction variable	Ethnic * Terrain ^1^ (2: Tibeto-Burman in Mountain land; 1: Others)	Categorical	EthnicTerrain	No unit

^1^ An interaction variable generated from Ethnic and Terrain. Regions belong to both Tibeto-Burman and mountain land are assigned with value 2, other regions are assigned with value 1 as references.

**Table 3:** Summary of the multiple logistic regression models on the risks for bacillary dysentery in southwest China.

Variable	Coefficient	OR	OR (95% CI)		*p*-Value	AUC ± SD	Kappa ± SD
Intercept	−24.557				<0.001		
DisFarm	−0.087	0.916	0.880	0.954	<0.001		
DisWater	−0.062	0.940	0.914	0.967	0.001		
GRP	−0.296	0.744	0.664	0.834	<0.001		
EthnicTerrain-2	1.112	3.059 ^1^	2.242	4.175	<0.001		
Forest-2	3.474	32.875 ^2^	13.154	82.188	<0.001		
Slope	−0.131	0.877	0.863	0.891	<0.001		
ElevationSD	0.542	1.721	1.535	1.931	<0.001		
RH_Ann	0.706	2.029	1.758	2.341	<0.001		
(RH_Ann) ^2^	−0.007	0.993	0.992	0.994	<0.001		
AT_Ann	0.169	1.184	1.135	1.236	<0.001		
BedNum-2	0.877	2.441 ^3^	1.178	5.062	<0.001		
BedNum-3	2.346	10.710	5.155	22.260	<0.001		
BedNum-4	2.981	20.234	9.187	44.586	<0.001		
Model						0.944 ± 0.004	0.75 ± 0.01

^1^ the odds ratio of EthnicTerrain-2 (Tibeto-Burman in Mountain land) is estimated in comparison to EthnicTerrain-1 (other situations); ^2^ the odds ratio of Forest-2 (forest coverage) is estimated in comparison to Forest-1 (non-forest coverage); ^3^ the odds ratio of BedNum-2, BedNum-3, BedNum-4 is estimated in comparison to BedNum-1 respectively.

**Table 4:** Accuracy assessment based on independent validation samples.

Predicted	Actual	
	P ^1^	A ^2^
**P**	113	16
**A**	14	111
Overall accuracy: 88.19%		

^1^ P: Presence; ^2^ A: Absence.

## Supplementary Materials

The following are available online at www.mdpi.com/1660-4601/14/7/782/s1, Figure S1: Example of a set of training samples (a total of 1000 training subsets were generated randomly in the bootstrapping logistic regression analyses.); Figure S2: Distribution of hotspot districts/counties year by year during the study period.
