# Supplementary material for: Environmental Drivers and Predicted Risk of Bacillary Dysentery in Southwest China
Source: Int J Environ Res Public Health. 2017 Jul 14;14(7):782. doi: 10.3390/ijerph14070782 (PMC5551220; doi:10.3390/ijerph14070782)
Supplement: Supplementary file 1 [file ijerph-14-00782-s001.pdf]

International Journal of
*Environmental Research and Public Health*

MDPI

## Supplementary Materials

# Environmental Drivers and Predicted Risk of Bacillary Dysentery in Southwest China

**Han Zhang, Yali Si, Xiaofeng Wang and Peng Gong**

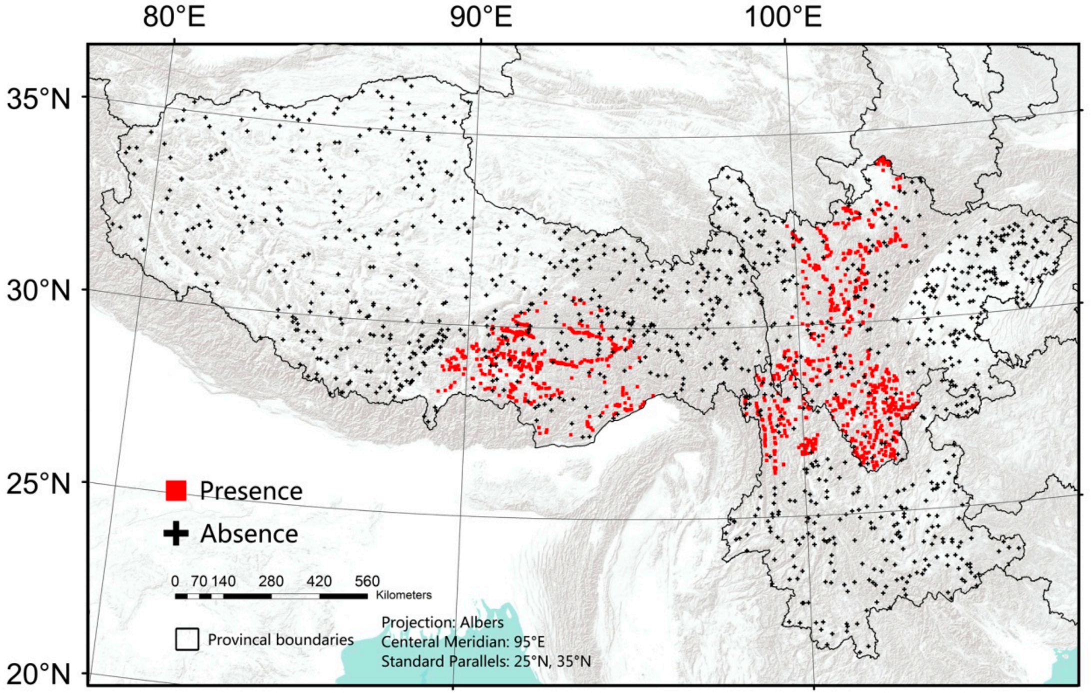


**Figure S1.** Example of a set of training samples (A total of 1000 training subsets were generated randomly in the bootstrapping logistic regression analyses.)

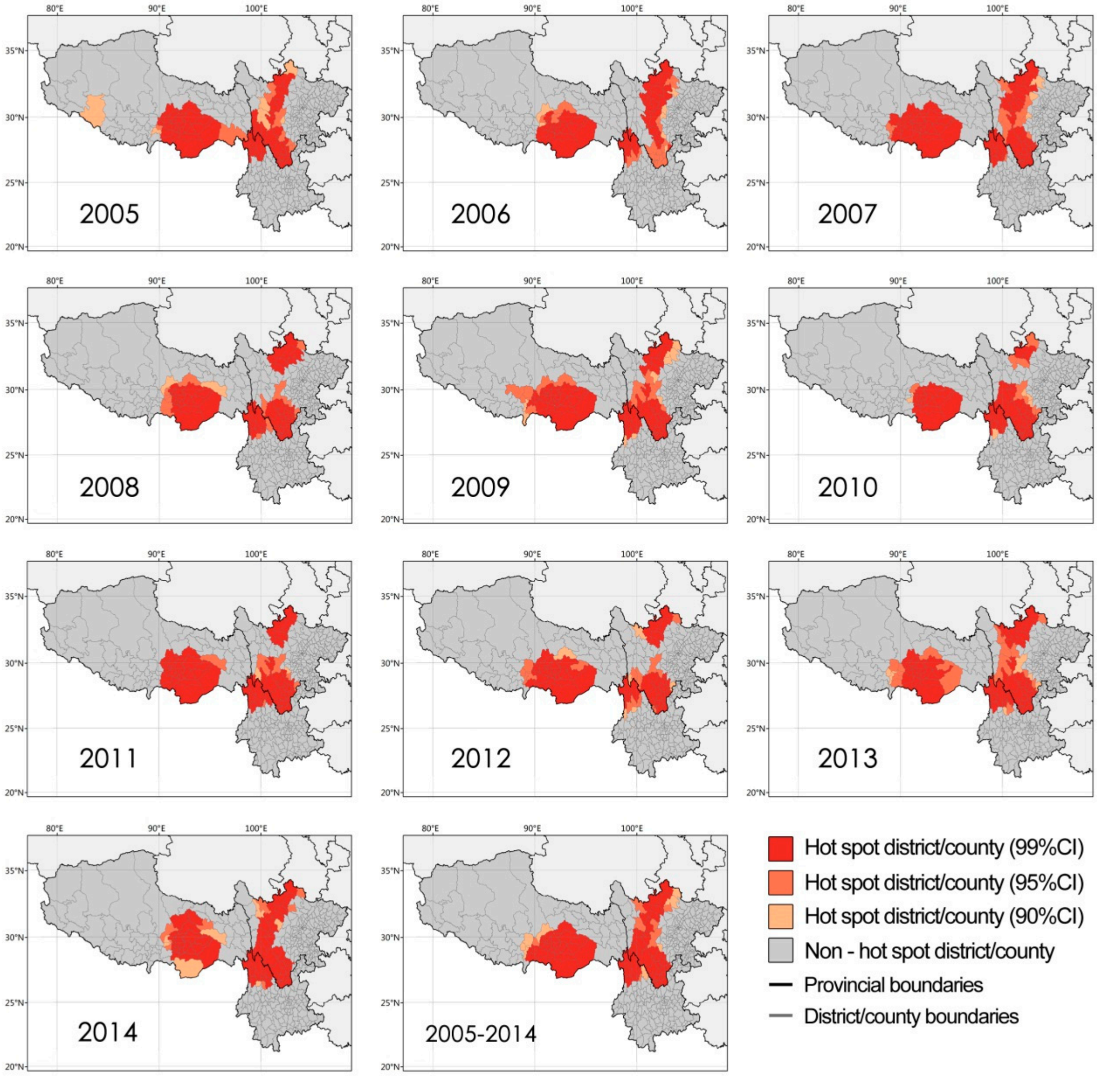


**Figure S2.** Distribution of hotspot districts/counties year by year during the study period.
